# Neural Correlates of the Time Course of the Waxing and Waning of Attention

**DOI:** 10.3389/fpsyg.2012.00377

**Published:** 2012-10-04

**Authors:** Paul Sajda

**Affiliations:** ^1^Department of Biomedical Engineering, Columbia UniversityNew York, NY, USA

When we are doing a task, such as driving our car, we often find ourselves slipping between intense task-engagement – e.g., we see an 18 wheel tractor trailer bearing down on us in our rear view mirror; and mind wandering – e.g., we are thinking about what is for dinner when we arrive home. This is often characterized as the waxing and waning of attention, and it has obvious implications for task performance. In the paper by Macdonald et al. (2011) the authors use a novel paradigm which attempts to tease apart subjective decision uncertainty and attentional state, while subjects perform a target detection task, as well as tie subjective attentional state to electroencephalographic (EEG) neural correlates.

The authors use a rapid visual serial discrimination (RSVP) task in which subjects must make a perceptual decision on whether they saw a target in a briefly presented image. In addition, subjects also report their confidence in their decision and how focused they were for the given trial. Interestingly, the authors found that the measured subjective responses of confidence and attentional state, though each was individually correlated with task performance, were not significantly correlated with one another. This indicates that perceived confidence and attentional state are more than just two sides of the same coin, and perhaps reflect, at least partially, different aspects of perceptual decision making.

To investigate this, the authors next explored correlating a variety of neural signals to subjective ratings. Specifically, they employed a set of analyses which considered three basic EEG phenomenological measures relevant to their paradigm – steady-state visually evoked potentials (SSVEP), pre-stimulus alpha power, and event related potentials (ERPs). The SSVEP has often been linked to attention and task-engagement (Muller et al., 2008) and the RSVP paradigm enables the authors to investigate modulations in SSVEP amplitude as they might reflect attentional waxing and waning toward the task. Surprisingly, neither amplitudes of the SSVEP nor early trial-averaged ERPs (i.e., the P1–N1 complex) varied significantly as a function of the subjects’ rating of their attentional state. Late ERPs that were differentially evoked by targets, namely the P300, were however modulated as a function of attentional state and confidence, specifically for target trials. One interesting possibility is that the P300 amplitude reflects the perceptual evidence available for a decision, with variability in attention and confidence reflecting the variability in perceptual representation of the target. In combination with the fact that the authors found no association between early perceptual EEG responses and attentional state rating, the P300 amplitude correlations suggest that effects of attentional state may occur at the decision level rather than the perceptual level. This is in agreement with previous findings (Ratcliff et al., 2009) which for a different perceptual decision making task, found temporally late, but not early, EEG components were informative for improving the estimation of the drift rate in a drift-diffusion model.

Next the authors investigated pre-stimulus alpha power and found that for trials in which subjects reported lowered attentional state, pre-stimulus alpha was higher. Particularly interesting was the time course of this relationship. Using a window based classification procedure, the authors found attentional state ratings smoothed over 7 min were most significantly correlated with pre-stimulus alpha. This suggests that the waxing and waning of attentional state occurs over relatively long periods in the task, which as the authors state, has practical implications for optimal information delivery.

There are a few differences between what Macdonald et al. report in terms of pre-stimulus alpha and previous studies featuring simple visual detection tasks. Most significantly is that Macdonald et al. do not see substantial differences between pre-stimulus alpha and correct vs. incorrect detection of targets, whereas pre-stimulus alpha power has often been reported as being greater preceding failed target detections (Ergenoglu et al., 2004; van Dijk et al., 2008; Busch et al., 2009). Additional work is needed to understand if this is idiosyncratic to the particular RSVP task.

In summary, the paper by Macdonald et al. yields new insights into how the subjective rating of our own attentional state and confidence are reflected in underlying neural correlates. There may be a bit of a chicken-and-egg problem on whether our conscious subjective ratings drive the changes in neural correlates or vice versa. Nonetheless, these types of studies not only provide us with a better characterization of how external events and our internal musings are reflected in neural oscillations and evoked responses, but ultimately begin to point toward possible brain computer interface (BCI) systems that may be able to help us manage our information overload (Sajda et al., 2010) – e.g., by making sure we keep our “mind on the road.”
